# Integrating bulk RNA-seq and ScRNA-seq to identify manganese metabolism-related subtypes and immunoregulatory mechanisms in liver hepatocellular carcinoma

**DOI:** 10.1515/biol-2025-1298

**Published:** 2026-04-29

**Authors:** Chunhui Liu, Wenqi Zhang, Wenzi Luo, Caifeng Zhang, Bo Zhang, Guozhi Zhang, Xiaotao Wang, Jianli Chen, Han Zhou

**Affiliations:** Department of General Surgery, North China University of Science and Technology Affiliated Hospital, Tangshan, Hebei, China; Department of Obstetrics and Gynecology, Tangshan Workers’ Hospital, Tangshan, Hebei, China; Department of Neurology, North China University of Science and Technology Affiliated Hospital, Tangshan, Hebei, China

**Keywords:** liver hepatocellular carcinoma, manganese metabolism-related genes, immunoregulatory mechanisms, ScRNA-seq

## Abstract

Liver hepatocellular carcinoma (LIHC) is an aggressive cancer associated with chronic liver disease, necessitating better biomarkers and therapies. Manganese, an essential trace element, regulates tumor development. Data from TCGA and GEO databases were analyzed to identify manganese metabolism-related genes (MMRGs). LIHC samples were classified into subtypes via consensus clustering. A prognostic model was developed using LASSO and multivariate Cox regression, then validated using ROC and survival analysis. Immune infiltration was assessed via ssGSEA and CIBERSORT, and cell communication was analyzed with single-cell data (GSE149614). Tumor Mutational Burden (TMB), drug sensitivity, and a nomogram were also evaluated. Two manganese metabolism-related subtypes were identified, with Cluster 1 showing better survival. A two-gene model (CEP55 and SPP1) reliably predicted poor prognosis in high-risk groups. The high-risk group exhibited distinct immune profiles, including increased immune infiltration, elevated checkpoint expression, and higher TIDE scores. Single-cell analysis revealed altered T cell communication. This study established manganese metabolism-related subtypes and a prognostic model for LIHC, providing insights into immunoregulation and cell communication to guide precision diagnosis and immunotherapy.

## 1 Introduction

Liver cancer ranks among the most prevalent malignancies globally and is a major contributor to cancer deaths worldwide [1]. It is not a single disease but rather a group of malignancies originating in the liver. Liver hepatocellular carcinoma (LIHC) is the predominant form of primary liver cancer – a highly aggressive malignant tumor whose development and progression are closely associated with chronic liver diseases and a cirrhotic background [2]. Approximately 80 % of cases occur in patients with cirrhosis, with major driving factors including chronic hepatitis B and C virus (HBV/HCV) infections [3], alcohol-related liver disease [4], and the increasingly prevalent non-alcoholic fatty liver disease (NAFLD) [5]. The pathogenesis of LIHC involves complex molecular processes, including genetic mutations, aberrant activation of oncogenic signaling pathways such as Wnt/β-catenin, and loss of tumor suppressor functions [6], 7]. Integrated bioinformatics analyses have been widely applied to identify key hub genes and potential therapeutic targets in HCC, providing a systematic framework for targeted therapy [8]. Moreover, dysregulated non-coding RNAs have also been reported to influence liver cancer progression through regulation of downstream targets, such as the miR-873/SOX4 axis [9]. Although surveillance of high-risk populations via ultrasound and alpha-fetoprotein (AFP) aims to achieve early detection, patients often present with symptoms such as abdominal pain and weight loss at advanced stages [10]. The treatment strategy for LIHC critically depends on tumor stage and liver function reserve: early-stage disease may be managed with curative approaches such as surgical resection, liver transplantation, and local ablation [11]. For advanced LIHC, systemic therapies represented by immune checkpoint inhibitors combined with anti-angiogenic agents have transformed the treatment landscape, setting a new benchmark for extending patient survival [12], 13]. Despite these significant advances, effective treatment options remain limited for many patients. There is an urgent need for novel biomarkers and therapeutic targets. Recent studies have identified regulatory axes such as miR-3130-5p/FDX1 as potential prognostic markers and therapeutic targets in HCC, highlighting the value of integrated bioinformatics and experimental validation [14].

Manganese, as an essential trace element, is present in very low concentrations in living organisms. However, its metabolic processes – including dietary absorption, transport through the bloodstream, and intracellular homeostasis regulation – are crucial for sustaining life activities [15], 16]. Manganese primarily functions as a metal cofactor for various enzymes, the most well-known of which is manganese superoxide dismutase (MnSOD) located in mitochondria. This enzyme plays a key role in the cellular antioxidant defense system by scavenging reactive oxygen species, thereby maintaining cellular and mitochondrial health [17], 18]. Liver cancer progression is often accompanied by oxidative stress and dysregulated apoptosis, which have been explored in experimental models such as HepG2 cells treated with plant-derived protein fractions [19]. Additionally, manganese significantly influences tumor initiation and progression. Tumors accumulate Mn^2+^, which triggers syndecan-1/β-integrin signaling and MMP-2/9 release, accelerating cancer-cell migration and invasion [20]. In pancreatic ductal adenocarcinoma, widespread down-regulation of manganese-import and retention genes reduces intracellular manganese levels, impairing MnSOD antioxidant capacity. This leads to redox-driven DNA damage and sustains YAP/TAZ oncogenic transcription, thereby accelerating tumor growth and immune evasion [21]. Mn^2+^ amplifies lipid ROS through a “Fenton-like reaction,” depletes glutathione (GSH), and inhibits GPX4, thereby inducing ferroptosis in tumor cells. Simultaneously, it activates the cGAS-STING and TLR4-NF-κB pathways, enhancing NK/T cell-mediated anti-tumor immunity [22]. Although evidence suggests that manganese plays a regulatory role in tumorigenesis, its specific molecular mechanisms in the development of LIHC still need to be further elucidated.

This study aims to integrate bulk and single-cell RNA sequencing data to establish manganese metabolism-related molecular subtypes, construct a robust prognostic model, and characterize the associated immunoregulatory mechanisms and cell communication networks in LIHC.

## 2 Materials and methods

### 2.1 Data collection

We acquired multi-omics data for LIHC from The Cancer Genome Atlas (TCGA) database (https://portal.gdc.cancer.gov/), which included mRNA expression profiles (50 normal and 342 tumor samples), mutation data, and clinical information. After excluding samples with a survival time of less than 30 days, 342 LIHC samples were retained for subsequent analysis. An independent validation set was obtained from the Gene Expression Omnibus (GEO) database (https://www.ncbi.nlm.nih.gov/geo/) under the accession number GSE14520 (*N* = 221). Single-cell sequencing data were sourced from the dataset GSE149614. Only primary tumor and adjacent non-tumor liver samples were included, and all other sample types were excluded, to maintain consistency with the TCGA-LIHC tumor-normal comparison. The single-cell data were used to support cell-type-level interpretation of the bulk RNA-seq findings. Furthermore, a list of 1,829 manganese metabolism-related genes (MMRGs) was directly adopted from a previously published study [21]. No additional screening or modification of the gene list was performed in the present study. Gene Ontology (GO) and Kyoto Encyclopedia of Genes and Genomes (KEGG) enrichment analyses were subsequently conducted to functionally characterize this gene set, with the results shown in Supplementary Figure 1.

### 2.2 Construction of manganese metabolism-related LIHC subtypes

The prognostic significance of MMRGs was assessed in the TCGA-LIHC cohort through univariate Cox regression analysis, with genes meeting a stringent threshold (*p* < 0.001) selected for subsequent clustering. Molecular subtyping of the samples was then performed using the “ConsensusClusterPlus” package, which defined two consensus clusters. Inter-subtype differential expression analysis, conducted with the “limma” package (|logFC| > 1 and FDR < 0.05), yielded a set of differentially expressed genes (DEGs).

### 2.3 Screening prognostic features to construct a prognostic model

The TCGA-LIHC dataset was randomly split into a training set (70 %) and two internal validation sets (30 % and 100 % of the cohort). In the training set, differential genes from the MMRG-related subtypes first underwent univariate Cox regression, and 169 genes with *P* < 0.05 that also passed the proportional hazards (PH) assumption test were retained as candidates. To prevent overfitting, Least Absolute Shrinkage and Selection Operator (LASSO) regression was performed using the “glmnet” package, where the optimal penalty parameter (lambda) was determined via cross-validation to refine the gene set. Subsequently, multivariate Cox regression on the LASSO-selected genes identified the final feature genes for the risk model. A risk score was computed for each patient based on the expression of these genes and their coefficients. Patients were stratified into high- and low-risk groups by the median score. Survival and time-dependent Receiver Operating Characteristic (ROC) curves (for 1, 3, and 5 years) were generated using the “survival” and “timeROC” packages to evaluate the model’s predictive performance, which was further validated in the two internal TCGA sets and the external GSE14520 cohort.

### 2.4 Immune infiltration analysis

Single-sample Gene Set Enrichment Analysis (ssGSEA) delineated differences in immune-related pathways and infiltrating immune cells. Stromal and immune constituents were assessed via the “ESTIMATE” package, while CIBERSORT enabled finer deconvolution of immune cell subsets. Inter-group differences in the expression of immune checkpoint molecules were statistically evaluated. The potential for immune evasion was inferred by computing the Tumor Immune Dysfunction and Exclusion (TIDE, http://tide.dfci.harvard.edu/) score, with its inter-group variation displayed in a violin plot.

### 2.5 Evaluation of independent prognostic factors

Integrating clinical parameters with the prognostic risk scores, univariate and multivariate Cox regression analyses were conducted, and the results were presented as forest plots. A nomogram was then developed using the “rms” package to predict 1-, 3-, and 5-year survival probabilities. The accuracy of the nomogram was assessed using calibration curves, and its potential as an independent prognostic factor was evaluated.

### 2.6 Pathway enrichment analysis of risk groups and subtypes in LIHC

We utilized the “limma” package to identify DEGs between the high- and low-risk groups in LIHC (criteria: |logFC| > 1 and p.adj < 0.05). Subsequently, the “clusterProfiler” package was applied to conduct multiple enrichment analyses, including Gene Set Enrichment Analysis (GSEA) for the risk groups, as well as GO and KEGG analyses on the up- and down-regulated DEGs from the risk comparison and the previously identified subtype DEGs.

### 2.7 Tumor mutational burden and drug sensitivity prediction

The analysis comprised two main parts: mutation profiling and drug discovery. First, Tumor Mutation Burden (TMB) was computed from the TCGA-LIHC mutation dataset. Subsequently, the mutation spectra of the top 20 genes were statistically analyzed and depicted in waterfall plots (“GenVisR” package) for the respective risk groups. Furthermore, for drug exploration, the CellMiner database (https://discover.nci.nih.gov/cellminer/) and DGIdb databases (https://www.dgidb.org) were leveraged to screen for drugs correlated with prognostic genes and to identify potential drug-target interactions. The IC50 values of candidate drugs were predicted using the “pRRophetic” package.

### 2.8 Processing and annotation of single-cell data

Single-cell data processing and clustering were performed using the Seurat package. Initial quality control filtered out cells with nCount_RNA < 200 or >30,000, nFeature_RNA < 200 or >6,000, or mitochondrial gene percentage (mt_percent) > 10 %. The data were then normalized using the ScaleData function. Principal component analysis (PCA) was conducted, and the top 30 principal components (PCs) were selected based on the ElbowPlot, capturing the majority of variance while avoiding overfitting. Unsupervised clustering was performed using the FindNeighbors and FindClusters functions with a resolution parameter set to 0.4, resulting in 24 distinct clusters. These clusters were subsequently annotated into 7 major cell types according to canonical marker genes.

### 2.9 Analysis of cell–cell communication

Cell–cell communication was inferred and analyzed using the CellChat R package. Normalized single-cell data with annotated cell types were used as input. The createCellChat and computeCommunProb functions were applied to quantify communication probabilities between clusters. Significant interactions were defined using a false discovery rate (FDR < 0.05). The aggregateNet function summarized the overall incoming and outgoing signaling patterns among clusters.

### 2.10 Quantitative real-time PCR

Total RNA was extracted from hepatocellular carcinoma cell lines and normal hepatic cell lines using the RNApure Tissue & Cell Kit with DNase I (CWBIO, Jiangsu, China) according to the manufacturer’s instructions. Reverse transcription was performed using the HiFiScript gDNA Removal RT Master Mix (CWBIO, Jiangsu, China). Quantitative real-time PCR was carried out using the MagicSYBR Mixture (CWBIO, Jiangsu, China). GAPDH was used as the endogenous reference gene for normalization. Relative gene expression levels were calculated using the 2−ΔΔCt method. All experiments were performed in triplicate. The primer sequences are listed in Table 1.

**Table 1:** Sequences of primers for qRT-PCR.

Gene name	Primer sequences (5′–3′)
CEP55	Forward: GCT​TCT​TTG​GAC​TTG​GCG​AC
	Reverse: CCT​CAA​GGA​CTC​GAA​TTT​TCT​CC
SPP1	Forward: AAA​CGC​CGA​CCA​AGG​AAA​AC
GPADH	Reverse: TGC​CTA​GGA​GGC​AAA​AGC​AA
	Forward: CTG​GGC​TAC​ACT​GAG​CAC​C
	Reverse: AAG​TGG​TCG​TTG​AGG​GCA​ATG

### 2.11 Statistical analysis

All statistical analyses and visualizations were conducted in R (v4.3.1) and Seurat (v4.3.0.1). Differences between two groups were assessed using the Wilcoxon test, with a *p*-value < 0.05 considered statistically significant. The “ggplot2” package was primarily used for data visualization.

## 3 Results

### 3.1 Manganese metabolism-related molecular cluster analysis

**Figure 1:**
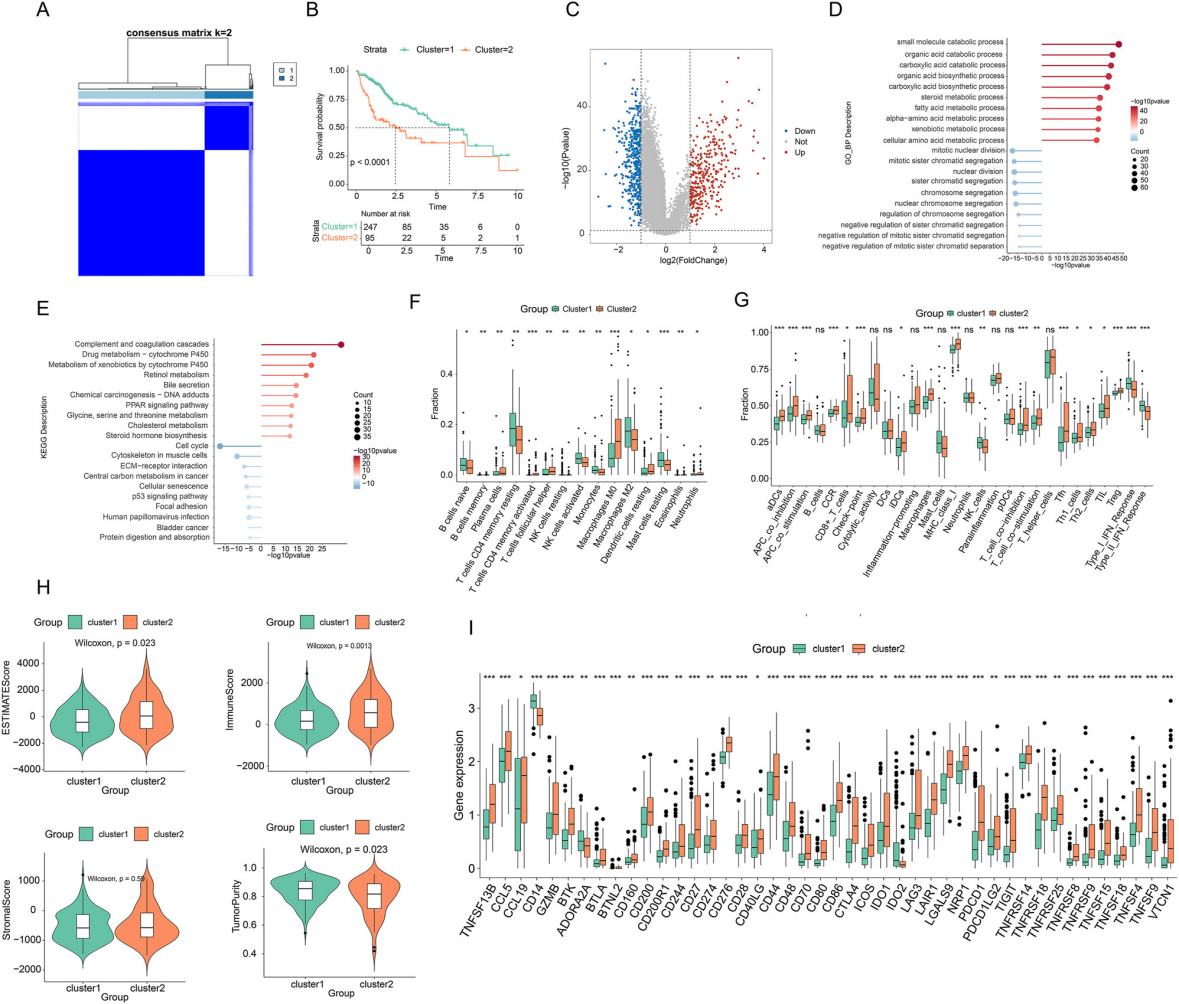
Identification of molecular clusters in LIHC. (A) Clustering heatmap based on MMRGs. (B) Survival curves of the 2 manganese metabolism-related molecular clusters. (C) Volcano plot of DEGs between clusters. (D) GO enrichment analysis of cluster-specific DEGs. (E) KEGG enrichment analysis of cluster-specific DEGs. (F) Box plot of immune cell type distribution across clusters. (G) Box plot of ssGSEA immune infiltration scores between clusters. (H) Violin plot of ESTIMATE score differences between the two clusters. (I) Box plot of immune checkpoint expression levels across clusters.

Based on MMRGs, TCGA-LIHC samples were stratified into two subtypes: Cluster 1 (*n* = 247) and Cluster 2 (*n* = 95) (Figure 1A). Kaplan–Meier analysis indicated that Cluster 1 was associated with significantly more favorable survival outcomes (Figure 1B). Comparative analysis identified 797 DEGs between the clusters (Figure 1C). Functional enrichment revealed distinct biological themes: GO analysis showed upregulated genes in Cluster 1 were involved in catabolic and biosynthetic processes (e.g., fatty acids, amino acids), while downregulated genes were linked to cell cycle and nuclear division (Figure 1D). Consistently, KEGG analysis highlighted enrichment of metabolic pathways (lipid, amino acid, xenobiotics) in Cluster 1, and cell cycle and p53 signaling pathways in Cluster 2 (Figure 1E). The immune microenvironment also differed substantially. CIBERSORT analysis revealed Cluster 1 was enriched in naive B cells and activated NK cells, whereas Cluster 2 had more plasma cells and M0 macrophages (Figure 1F). ssGSEA further confirmed that most immune functions and infiltration scores were elevated in Cluster 2 (Figure 1G). Accordingly, ESTIMATE analysis showed Cluster 2 possessed significantly higher Immune, Stromal, and ESTIMATE scores but lower Tumor Purity (Figure 1H). Concordantly, immune checkpoint gene expression was markedly upregulated in Cluster 2 (Figure 1I).

### 3.2 Development and assessment of a prognostic signature

**Figure 2:**
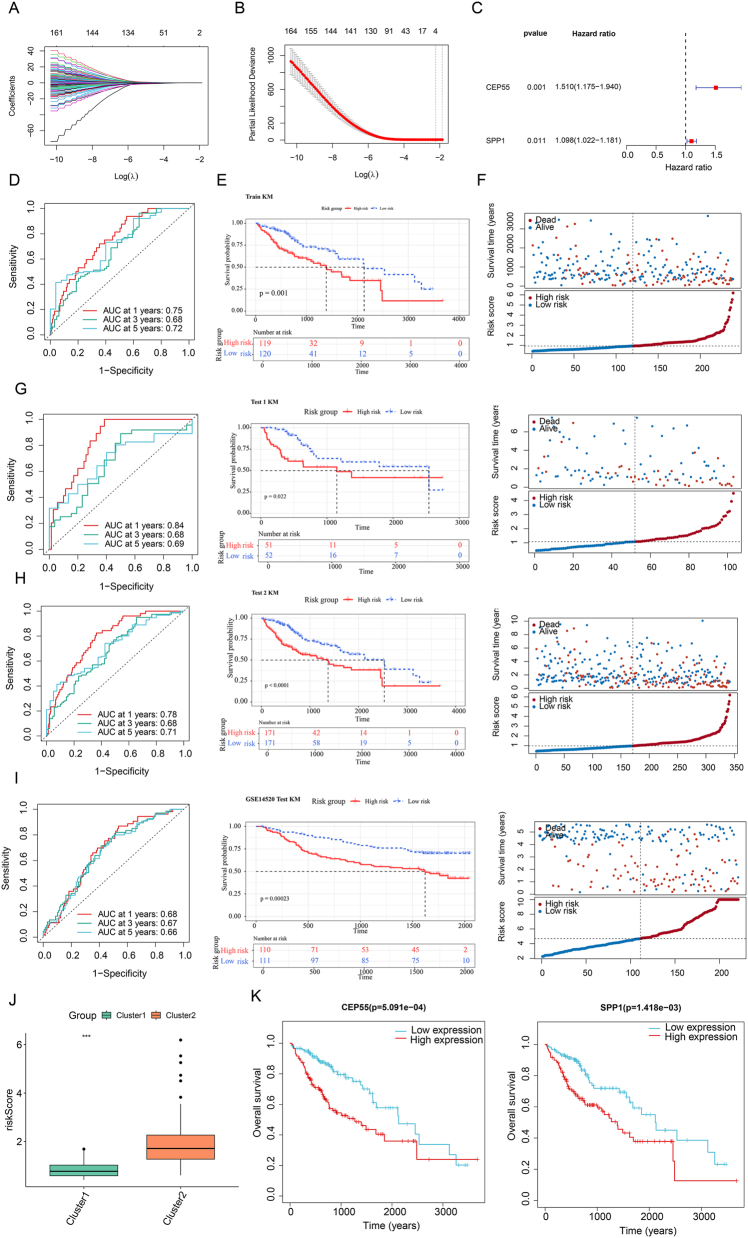
Prognostic model development and validation. (A) Coefficient distribution plot generated for the log(*λ*) sequence in the LASSO model. (B) LASSO coefficient profile of the LASSO Cox analysis. (C) Forest plot of the multivariate regression analysis. (D) AUC curves for 1-, 3-, and 5-year predictions by the model in the training set. (E) Survival curves of overall survival for high-risk and low-risk groups in the training set. (F) Survival status distribution plot and risk score distribution plot in the training set. Validation cohorts Test 1 (G), Test 2 (H), and GSE14520 (I): ROC curves, Kaplan–Meier survival curves, survival status distribution plots, and risk score distribution plots. (J) Box plot of risk scores across clusters. (K) Kaplan–Meier curves of the characteristic genes.

From the cluster-specific DEGs, univariate Cox regression in the TCGA training set identified 169 prognostic candidate genes (*p* < 0.05; Supplementary Table 1). Subsequent LASSO regression narrowed these down to four feature genes (Figure 2A and B), and a final multivariate Cox regression selected two genes (CEP55 and SPP1) to construct the prognostic model (Figure 2C).

The risk score was calculated as:

Riskscore=0.41210×CEP55+0.09370×SPP1.

Based on the prognostic model, each patient was assigned a risk score. In the training set, the model demonstrated robust predictive power for 1-, 3-, and 5-year survival (AUCs: 0.75, 0.68, 0.72; Figure 2D), with the high-risk group exhibiting significantly poorer outcomes (Figure 2E). The risk score distribution and survival status were visualized (Figure 2F). Concordantly, the model’s efficacy was validated across three independent cohorts (30 % TCGA, 100 % TCGA, and GSE14520), where the time-dependent Area Under the Curves (AUCs) for survival prediction all exceeded 0.66, and survival analysis uniformly confirmed the adverse prognosis of the high-risk group (Figure 2G–I). Furthermore, risk scores were significantly elevated in Cluster 2 (Figure 2J), and high expression of the two signature genes (CEP55 and SPP1) was individually associated with poorer survival (Figure 2K).

### 3.3 Independent prognostic factor analysis

**Figure 3:**
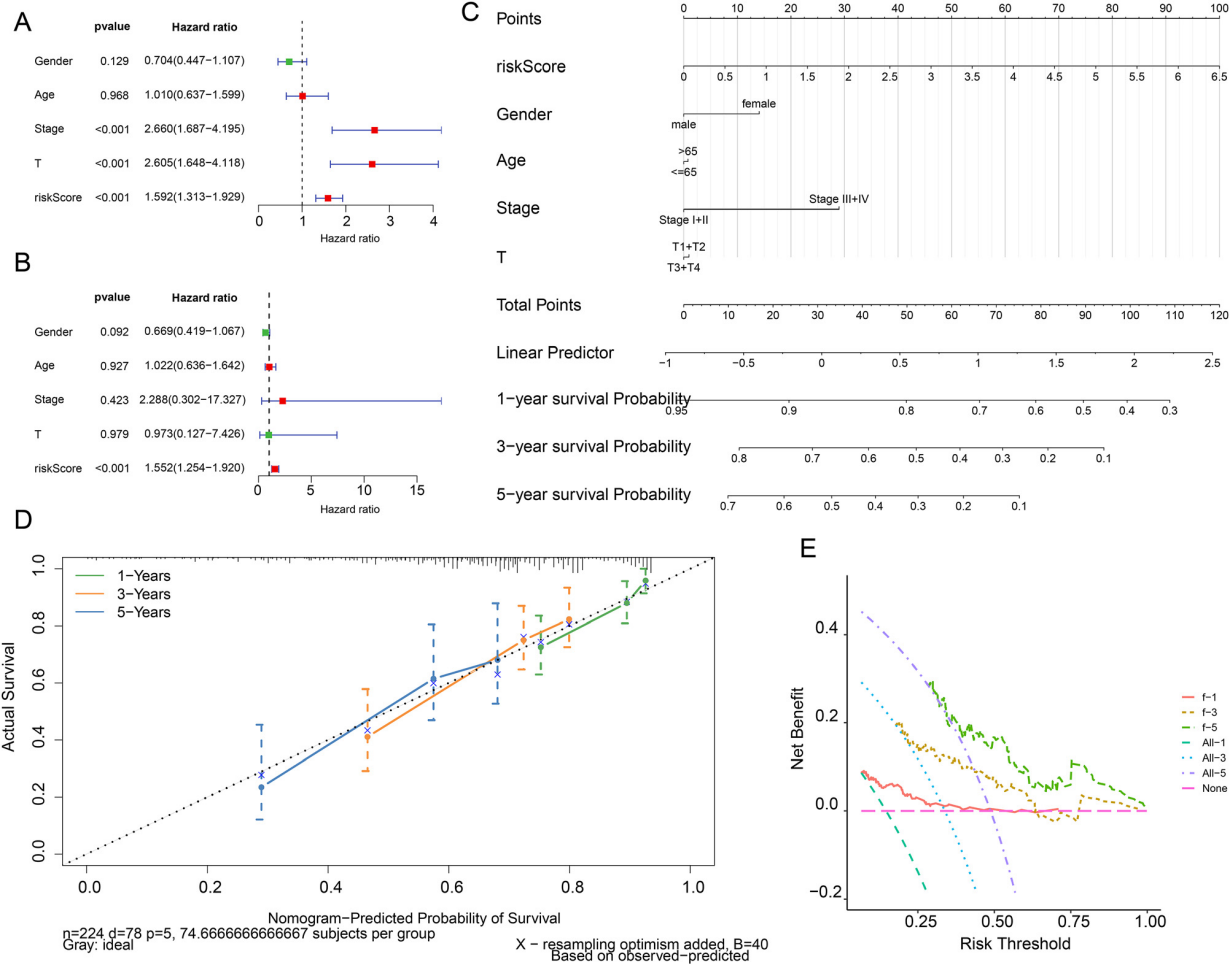
Nomogram survival model: development and validation. (A) Univariate and (B) multivariate Cox regression analyses of prognostic factors. (C) Nomogram integrating the risk score and clinical parameters for survival prediction. (D) Calibration curves and (E) DCA for the nomogram at 1, 3, and 5 years.

Univariate Cox regression identified Stage, T stage, and riskScore as significant prognostic factors (Figure 3A), while multivariate analysis confirmed riskScore as an independent predictor (Figure 3B). A nomogram integrating these clinical parameters was constructed to estimate individual survival probability (Figure 3C). The model demonstrated high predictive accuracy, evidenced by excellent concordance between predictions and actual outcomes in the 1-, 3-, and 5-year calibration curves (Figure 3D). Furthermore, decision curve analysis (DCA) confirmed the nomogram’s robust clinical utility for survival prediction at all three time points (Figure 3E).

### 3.4 Enrichment analysis of high-/low-risk groups

**Figure 4:**
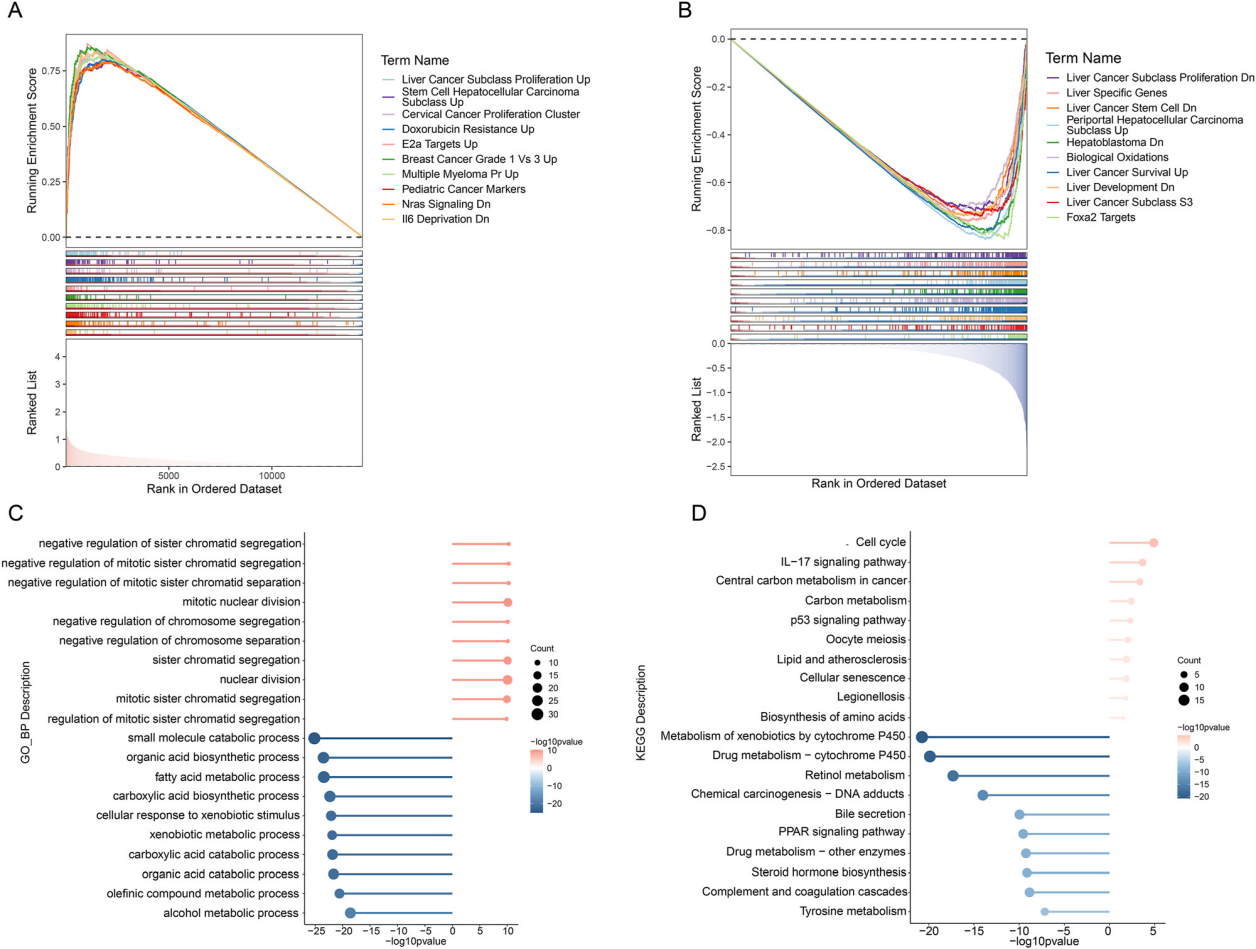
Enrichment analysis between high-/low-risk groups. GSEA for the high- (A) and low-risk (B) groups. Functional enrichment of DEGs: GO (C) and KEGG (D) terms.

GSEA revealed distinct pathway activities between risk groups. The high-risk group showed strong positive enrichment in tumorigenesis and proliferation pathways (e.g., Liver Cancer Subclass Proliferation; Figure 4A). Conversely, the low-risk group exhibited significant negative enrichment in pathways governing liver-specific functions, cellular metabolism, and development, indicating their suppression (Figure 4B). GO analysis of DEGs showed upregulation of mitotic and chromosomal processes, and downregulation of metabolic processes like fatty acid catabolism (Figure 4C). Similarly, KEGG analysis indicated upregulated genes were involved in cell cycle and IL-17 signaling, while downregulated genes were enriched in cytochrome P450 metabolism and bile secretion (Figure 4D).

### 3.5 Immune landscape analysis in high-/low-risk group

**Figure 5:**
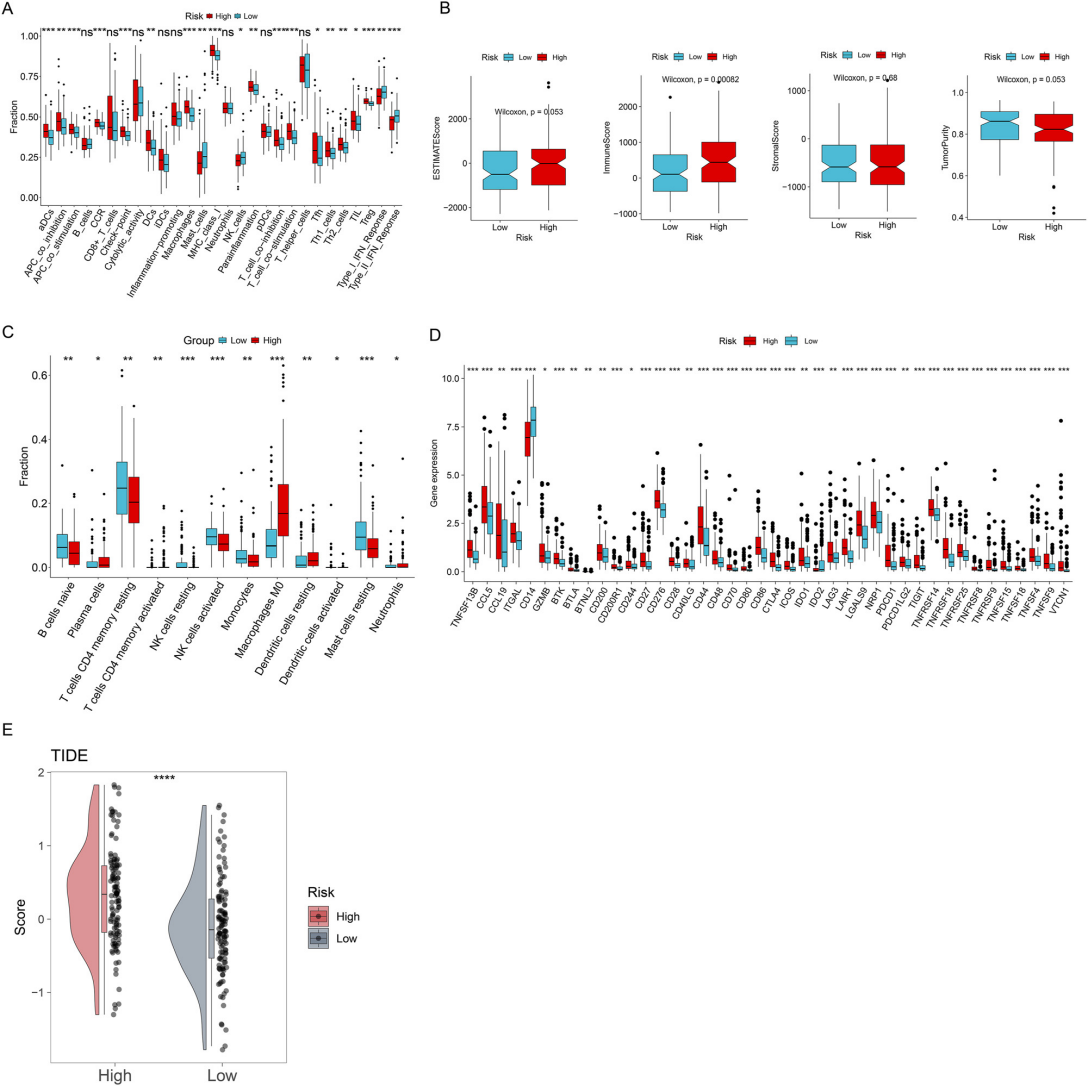
Immune microenvironment characterization. (A) ssGSEA immune infiltration scores. (B) ESTIMATE-derived scores and tumor purity. (C) CIBERSORT immune cell fractions. (D) Immune checkpoint gene expression. (E) TIDE score distribution.

Analysis using the ssGSEA algorithm demonstrated that the high-risk group exhibited significantly higher scores for immune-related functions and infiltration of CD8^+^ T cells, dendritic cells, Th1/Th2 cells, and macrophages, whereas the low-risk group showed elevated infiltration of NK cells and mast cells (Figure 5A). ESTIMATE analysis revealed significantly higher Immune and ESTIMATE scores in the high-risk group, which conversely displayed lower Tumor Purity (Figure 5B). CIBERSORT algorithm analysis indicated that the high-risk group had significantly increased infiltration of plasma cells and M0 macrophages, while the low-risk group exhibited higher levels of naive B cells, activated NK cells, and resting mast cells (Figure 5C). Notably, the results from ssGSEA, ESTIMATE, and CIBERSORT were largely consistent, supporting the robustness of our immune infiltration analysis. Specifically, high-risk patients consistently exhibited increased infiltration of macrophages, CD8^+^ T cells, and dendritic cells by ssGSEA and CIBERSORT, whereas low-risk patients showed higher levels of NK cells and mast cells. ESTIMATE analysis further confirmed that high-risk patients had elevated Immune and ESTIMATE scores, consistent with the patterns observed in ssGSEA and CIBERSORT. Although each algorithm emphasizes different aspects – ssGSEA highlights functional immune signatures, CIBERSORT quantifies specific immune cell fractions, and ESTIMATE provides overall immune activity – the concordance among these approaches reinforces the reliability of our findings. Additionally, the expression levels of immune checkpoint genes (Figure 5D) and the TIDE score (Figure 5E) were both markedly elevated in the high-risk group.

### 3.6 Genomic alteration and therapeutic response

**Figure 6:**
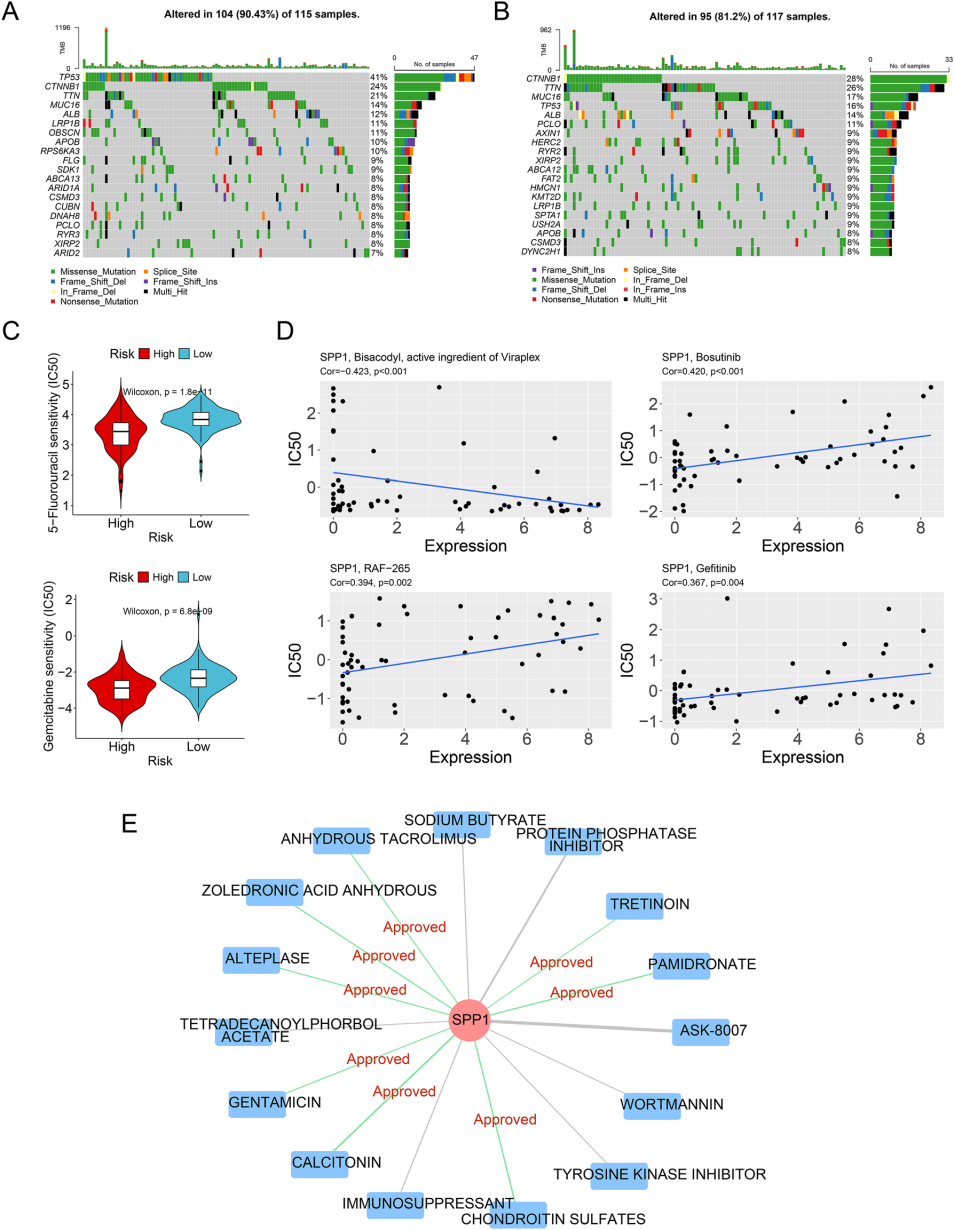
Analysis of mutational profiles and therapeutic relevance. Mutational landscape of the top 20 genes in the high-risk (A) and low-risk (B) groups. (C) IC50 to two chemotherapeutic agents across risk groups. (D) Scatter plot showing the correlation between signature gene expression and drug activity from CellMiner. (E) Network of candidate drugs interacting with the prognostic signature genes, sourced from DGIdb.

Analysis of TCGA mutation data revealed that TMB was comparable between risk groups. The top 20 mutated genes, including TP53, TTN, CTNNB1, and MUC16, were frequently altered in both cohorts (Figure 6A and B). Drug sensitivity analysis indicated that patients in the high-risk group were more sensitive to 5-Fluorouracil and Gemcitabine, as evidenced by their lower predicted IC50 values (Figure 6C). CellMiner analysis demonstrated significant positive correlations of RAF-265 (cor = 0.394), Bosutinib (cor = 0.420), and Gefitinib (cor = 0.367) with SPP1 expression, while Bisacodyl (cor = −0.423) showed a significant negative correlation with SPP1 (Figure 6D). The DGIdb database was utilized to identify drugs interacting with SPP1 (Figure 6E).

### 3.7 Preprocessing and characterization of single-cell transcriptomic data

**Figure 7:**
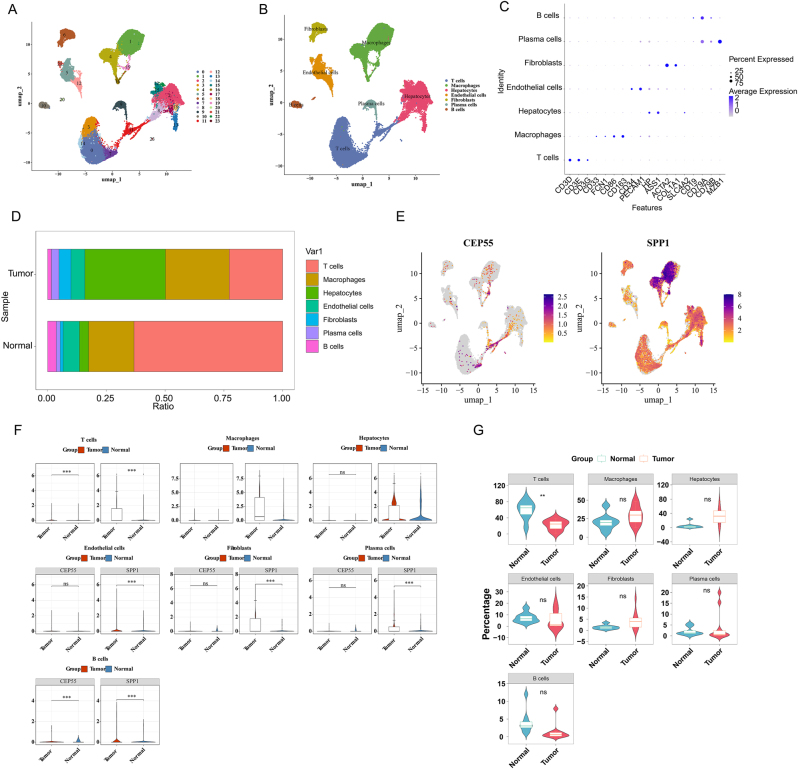
Single-cell classification and annotation. (A) Cell clustering analysis results. (B) Cell annotation results. (C) Expression of marker genes in annotated cells. (D) Bar plot of cellular proportions in tumor and normal groups. (E) Projection of diagnostic genes across cell clusters. (F) Expression patterns of prognostic genes in different cell types between tumor and normal groups. (G) Violin plot showing differences in cellular proportions between tumor and normal groups.

Integrated single-cell data comprising 55,501 cells were categorized into 23 distinct clusters (Figure 7A). Using standard marker genes, these cells were classified into 7 biologically defined cell types: Fibroblasts (ACTA2, COL1A1); Macrophages (CD86, CD163, FCN1, CD33); T cells (CD3D, CD3E, CD3G); Endothelial cells (CD34, PECAM1); Hepatocytes (HP, ASS1); Plasma cells (AZB1); and B cells (CD19, CD79A) (Figure 7B and C). Comparative analysis of cellular composition revealed significant differences between the normal and tumor groups. Comparative analysis revealed a distinct cellular landscape: tumor samples were enriched with Macrophages and Hepatocytes, whereas normal tissues had a higher proportion of T cells (Figure 7D). Expression analysis showed that CEP55 was expressed at low levels, primarily in Macrophages and Hepatocytes, while SPP1 was highly and specifically expressed in Macrophages (Figure 7E). Although both diagnostic genes showed differential expression in T and B cells (Figure 7F and G), only the proportion of T cells was significantly altered between tumor and normal conditions, designating it as the pivotal cell type for subsequent analysis.

### 3.8 Cell–cell communication analysis

**Figure 8:**
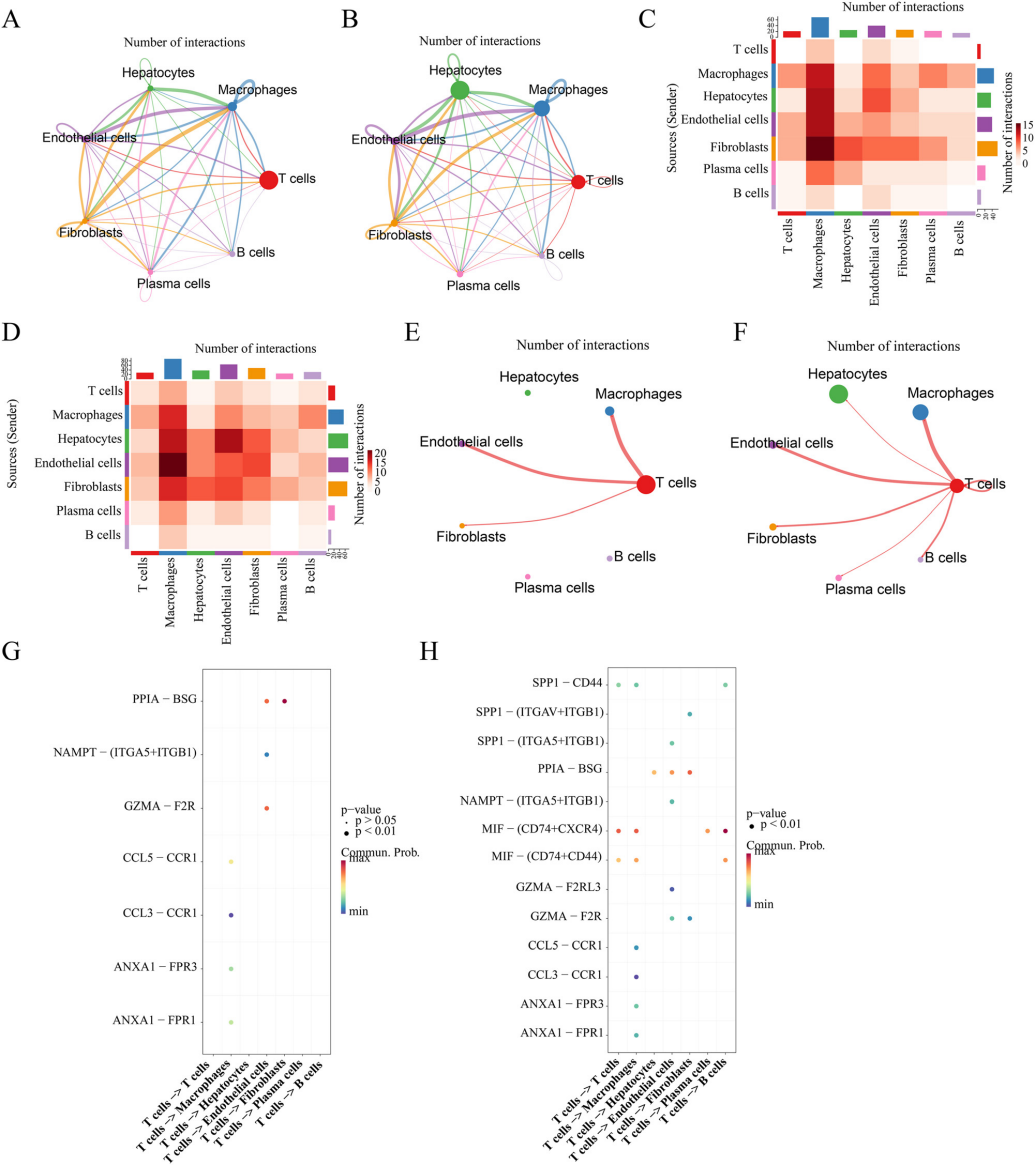
Cell–cell signaling communication analysis. CellChat circular plots for the (A) normal and (B) tumor groups. Heatmaps of ligand-receptor interactions in the (C) normal and (D) tumor groups. Communication networks of T cells in the (E) normal and (F) tumor groups. Bubble plots of T cell-specific interactions in the (G) normal and (H) tumor groups.

In both normal and tumor groups, Fibroblasts, Endothelial cells, and Hepatocytes functioned as central hubs in the cell communication network, participating in the highest number of interactions (Figure 8A and B). In the normal group, Fibroblasts as senders exhibited the strongest signaling output to Macrophages (Figure 8C). In the tumor group, Endothelial cells as senders demonstrated the most robust signaling output to Macrophages (Figure 8D). While T cells did not exhibit any ligand-receptor interactions with B cells, Plasma cells, or Hepatocytes in the normal group, they demonstrated evident ligand-receptor interactions with these cell types in the tumor group (Figure 8E and F). In the normal group, T cells engaged in multiple receptor-ligand interactions with macrophages, endothelial cells, and fibroblasts. The PPIA-BSG pair showed the highest communication probability between T cells and fibroblasts (Figure 8G). In tumor group, there are extensive receptor-ligand interactions between T cells and all types of cells, among which MIF-(CD74+CXCR4) shows the highest communication probability between T cells and B cells (Figure 8H).

### 3.9 Validation of prognostic gene expression by qRT-PCR

**Figure 9:**
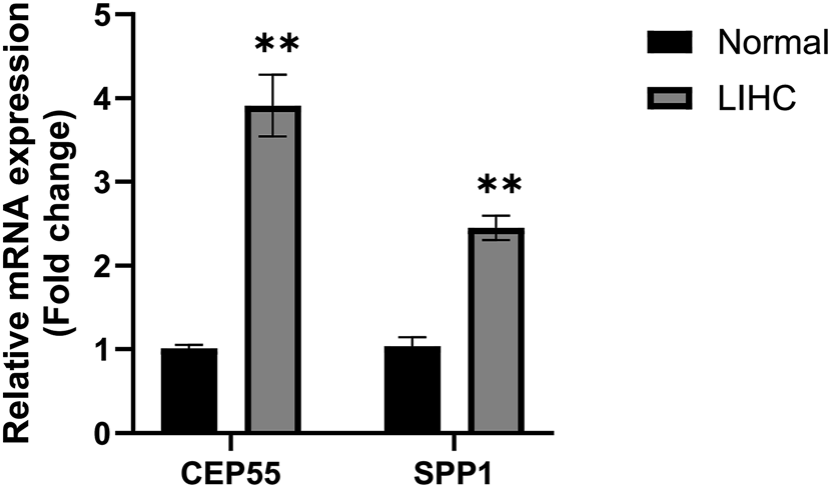
Validation of prognostic gene expression by qRT-PCR. qRT-PCR analysis of CEP55 and SPP1.

To validate the expression of the identified prognostic genes, qRT-PCR analysis was performed. The results showed that CEP55 and SPP1 were significantly upregulated in HCC cell lines compared with normal hepatocyte cell lines (Figure 9), which was consistent with the bioinformatics analysis. These findings further support the potential of CEP55 and SPP1 as prognostic biomarkers in hepatocellular carcinoma.

## 4 Discussion

This study elucidates the regulatory role of MMRGs in the pathogenesis of hepatocellular carcinoma and the tumor microenvironment. By integrating and analyzing bulk and single-cell RNA-seq data, we comprehensively delineated molecular clusters, constructed a reliable prognostic signature, and explored associated immunoregulatory mechanisms and intercellular communication dynamics. This comprehensive approach not only identified a novel gene-based prognostic model with validated clinical value but also revealed the interactions between manganese metabolism, tumor progression, and the complex immune landscape of LIHC. These findings lay the groundwork for a deeper understanding and provide potential avenues for precision diagnosis and the optimization of immunotherapy.

The 2 signature genes (CEP55 and SPP1) identified play a critical role in LIHC. CEP55 is a gene closely associated with cell division, playing a critical role particularly in the final stage of mitosis [23], 24]. It encodes the centrosome-associated protein 55, which is primarily involved in regulating cytokinesis, ensuring the normal and successful completion of cell division [25]. CEP55 is highly expressed in various cancers, such as breast cancer, lung cancer, liver cancer, kidney cancer, and colorectal cancer [26]. Its elevated expression is often closely correlated with higher tumor stages, increased invasiveness, and poorer prognosis [27]. This finding is consistent with the results observed in our current study, where high expression of CEP55 is associated with an unfavorable prognosis. In cancer cells, CEP55 overexpression interferes with midbody abscission and cytokinesis, leading to chromosomal mis-segregation and multinucleation, thereby directly inducing genomic instability and providing a basis for mutations that facilitate rapid evolution and the acquisition of drug resistance in cancer cells [28]. In hepatocellular carcinoma, CEP55 physically binds to and enhances the phosphorylation of JAK2, persistently activating the JAK2–STAT3–MMP2/9 signaling axis. This significantly promotes tumor cell migration, invasion, and metastatic potential, and is closely associated with an unfavorable prognosis in patients [29]. The SPP1 (Secreted Phosphoprotein 1) gene encodes a multifunctional glycosylated and phosphorylated acidic protein, also known as osteopontin [30]. It was initially identified as a key component in the bone matrix, but its functions are extensive, participating in various physiological and pathological processes. SPP1 influences cancer progression through multiple mechanisms, particularly in modulating the tumor microenvironment [31], 32]. In pancreatic ductal adenocarcinoma, SPP1 secreted by epithelial tumor cells acts as a critical messenger, activating the CD61 receptor on stromal cell surfaces, which in turn initiates the BMP2-GREM1 signaling axis, forming a bidirectional regulatory circuit [33]. This network collectively determines the invasive characteristics of the tumor and drives its malignant progression. In a colorectal cancer liver metastasis model, SPP1-stimulated production of CXCL12 by cancer-associated fibroblasts suppresses the infiltration of CD8^+^ T cells, leading to the formation of an immunosuppressive microenvironment [34]. Furthermore, in hepatocellular carcinoma (HCC), SPP1 acts as a “metabolic switch” that activates fatty acid metabolism pathways, driving tumor cells to acquire malignant traits such as proliferation, migration, and invasion, and even contributing to drug resistance [35]. CEP55 and SPP1 collectively drive the malignant progression and poor prognosis of cancer, particularly LIHC. Consistently, our single-cell analysis showed that CEP55 was mainly expressed in hepatocytes and macrophages, while SPP1 was highly expressed in macrophages. Moreover, tumor samples exhibited higher proportions of these cell types compared with normal tissues, supporting a role for CEP55 and SPP1 in tumor progression and modulation of the immune microenvironment in LIHC. Although the direct mechanistic link between CEP55/SPP1 and manganese metabolism remains to be fully elucidated, our GO and KEGG enrichment analyses indicate that manganese metabolism-related genes are significantly involved in oxidative stress response, metal ion homeostasis, and hypoxia-related pathways (Supplementary Figure 1). CEP55 may influence tumor progression through ROS-mediated pathways sensitive to manganese-dependent enzymes such as MnSOD. SPP1, as a secreted multifunctional protein, participates in metabolic reprogramming and tumor microenvironment modulation, potentially affecting manganese-associated redox balance and immune regulation. These observations suggest that CEP55 and SPP1 may play important roles in manganese-regulated tumorigenesis and remodeling of the immune microenvironment in LIHC.

The high-risk group exhibits higher scores for immune-related functions and immune cell infiltration in macrophages. Studies have shown that macrophages directly mediate tumor immune escape by upregulating bidirectional crosstalk between B7 family ligands and CD28 family receptors, thereby blocking T-cell costimulatory signals and reinforcing immune checkpoint inhibition [36]. Additionally, the TIDE score in the high-risk group is significantly higher than that in the low-risk group, suggesting a greater potential for immune escape or immune dysfunction [37], 38]. Research indicates that the upregulation of immune checkpoint expression enables tumor cells to systematically shut down T-cell costimulatory pathways by simultaneously enhancing multiple inhibitory signals such as PD-L1, CTLA-4, LAG-3, and TIM-3, thereby constructing a “multi-node” immune evasion network [39], 40]. The direct consequences of this include functional exhaustion and reduced tumor infiltration of CD8^+^ T cells, lower response rates to immune checkpoint inhibitor (ICB) therapy, and poorer patient prognosis [41], 42]. These factors collectively create a complex, inflammation-driven immunosuppressive microenvironment, ultimately leading to worse clinical outcomes.

The reprogramming of cellular communication networks in tumors highlights the role of endothelial cells in immune regulation. By expressing PD-L1, secreting chemokines, and regulating adhesion molecules, they determine T cell infiltration and function, serving as critical “gatekeepers” of tumor immune escape [43]. Notably, newly identified ligand-receptor interactions between T cells, B cells, and hepatocytes in tumors, such as the MIF-(CD74+CXCR4) axis, play a significant role in tumorigenesis. Studies have shown that epithelial-derived MIF in colorectal cancer autocrinally activates the MIF-CD74 circuit, which induces VEGF expression via the PI3K/AKT pathway, directly accelerating tumor cell proliferation and stimulating angiogenesis [44].

This study successfully integrated bulk RNA sequencing and single-cell RNA sequencing data to construct a reliable prognostic model based on MMRGs, and provided an in-depth characterization of molecular clusters, the complex immune microenvironment, and cell communication networks in LIHC. However, several key limitations remain. The primary limitation is the lack of experimental validation. Although the model genes CEP55 and SPP1 were screened and show a strong association with poor prognosis, their specific biological functions, molecular regulatory mechanisms, and potential as therapeutic targets in LIHC cell lines or animal models have not yet been confirmed through *in vivo* or *in vitro* functional experiments. Secondly, all analytical results in this study, including molecular subtyping, prognostic model construction, and immune landscape analysis, rely solely on existing retrospective data from public databases (TCGA and GEO). In addition, drug sensitivity analyses were performed using the CellMiner database. While these results suggest potential associations between SPP1 expression and therapeutic response, they are computational predictions that do not account for *in vivo* tumor complexity, including cellular heterogeneity, microenvironmental influences, and pharmacokinetic factors. Therefore, future fundamental and clinical experimental studies are necessary to address these limitations and ultimately facilitate the clinical translation of the model and key pathways.

## 5 Conclusions

By integrating bulk and single-cell RNA sequencing, this study established manganese metabolism-related clusters and a reliable prognostic model for LIHC, providing insights into associated immunoregulatory mechanisms and cell communication dynamics to guide precision diagnosis and immunotherapy optimization.

## Supplementary Material

Supplementary Material

Supplementary Material

Supplementary Material
