# Supplementary material for: Integrating bulk RNA-seq and ScRNA-seq to identify manganese metabolism-related subtypes and immunoregulatory mechanisms in liver hepatocellular carcinoma
Source: Open Life Sci. 2026 Apr 29;21(1):20251298. doi: 10.1515/biol-2025-1298 (PMC13127686; doi:10.1515/biol-2025-1298)
Supplement: Supplementary file 1 — Supplementary Material [file j_biol-2025-1298_suppl_001.pdf]

Supplementary Table 1. Univariate regression analysis results (p<0.05)

gene	HR	HR.95L	HR.95H	pvalue
SLC27A5	0.8851425	0.7895085	0.9923607	0.0364905
CEP55	1.725547	1.3776901	2.1612354	2.04E-06
MPZL1	1.4736797	1.1369331	1.9101667	0.0033948
LIMK1	1.37359	1.0761255	1.7532801	0.0107986
LMNB2	1.5927876	1.2324529	2.0584741	0.0003749
MAPRE1	1.5366033	1.1746995	2.0100032	0.0017184
VASP	1.3157016	1.0125368	1.7096373	0.0400534
NAP1L1	1.6148956	1.2076829	2.1594145	0.0012257
PNMA1	1.4483311	1.1545247	1.8169062	0.0013642
ARHGEF2	1.4099887	1.0994321	1.8082682	0.0067944
ELF4	1.3942627	1.0813773	1.7976782	0.0103667
ALDH2	0.7129983	0.5711483	0.890078	0.0028006
HAO1	0.8712166	0.7790766	0.9742539	0.0156358
SLC1A5	1.3584732	1.1758449	1.5694667	3.20E-05
BDH1	0.7575471	0.62703	0.9152314	0.0040009
IMPDH1	1.4105205	1.1785827	1.6881023	0.000175
HOMER3	1.3005148	1.0067504	1.679998	0.0442763
DBN1	1.1919326	1.0008911	1.4194385	0.0488433
PLEKHB2	1.4154764	1.0858609	1.8451476	0.0101996
MMD	1.3863047	1.1227846	1.7116735	0.0023926
UPB1	0.8445336	0.7501382	0.9508075	0.0052044
RBP4	0.8543234	0.7648386	0.9542778	0.0052873
SLC38A1	1.3620841	1.1413428	1.6255178	0.0006137
RGN	0.8450477	0.7266404	0.9827497	0.0288242
LAMB1	1.3109658	1.0685167	1.6084272	0.0094551
SLC16A3	1.4480474	1.2094869	1.7336617	5.56E-05
PPT1	1.6289594	1.2516848	2.1199496	0.0002833
CA9	1.121036	1.0160439	1.2368773	0.0227744
ENO2	1.2660133	1.0431029	1.5365596	0.0169854
MARCKS	1.328943	1.0669736	1.6552327	0.0111278
CYP27A1	0.8583094	0.7460149	0.9875072	0.0327049
CALU	1.5895171	1.2084095	2.0908181	0.0009214
CFHR2	0.8615995	0.7783022	0.9538116	0.0040848
SFXN3	1.3177485	1.0574945	1.642052	0.0139735
HSD17B6	0.8854879	0.8073598	0.9711764	0.0098643
MARCKSL1	1.3728407	1.1472169	1.6428382	0.0005418
ST6GALNAC[illegible]	1.4643411	1.1615603	1.8460469	0.0012503
YWHAZ	1.4513938	1.1312785	1.8620915	0.0033875
S100A11	1.2082632	1.0501681	1.3901584	0.0081905
EHHADH	0.8569858	0.7411491	0.990927	0.0372532
EGLN3	1.3252189	1.0862038	1.6168284	0.0055231
CPB2	0.8508247	0.7430141	0.9742785	0.0194439
SEC14L2	0.8526387	0.744694	0.9762301	0.0209828
KCTD17	1.2485863	1.0570602	1.4748145	0.0089723
SLC10A3	1.6061129	1.2295596	2.0979857	0.0005089
HAGH	0.740863	0.587053	0.9349716	0.0115272
SMOX	1.3574504	1.105462	1.6668793	0.0035347
PLAUR	1.3173401	1.0461344	1.6588547	0.0191064
PFKFB3	1.1782248	1.0025819	1.3846387	0.0464511

RGS2	1.1434995	1.004043	1.3023258	0.0433038
NT5DC2	1.3207718	1.1046787	1.5791362	0.0022724
SLC39A6	1.3262261	1.0394165	1.6921759	0.0231523
SERPING1	0.8136878	0.6630522	0.9985456	0.0483933
ATP1B3	1.4624242	1.196534	1.7873997	0.0002052
SLC7A7	1.3452678	1.0481524	1.7266053	0.0198427
ABAT	0.8619659	0.7509207	0.9894324	0.0347772
B4GALT5	1.5170298	1.1960372	1.9241704	0.0005909
HPX	0.8715838	0.7962049	0.9540991	0.0029006
TNFRSF21	1.1821908	1.0042439	1.391669	0.044342
SLC2A1	1.6080747	1.2991373	1.990478	1.28E-05
UAP1L1	1.2386174	1.0122717	1.5155743	0.03767
GCDH	0.7351371	0.5674199	0.9524278	0.019866
HK2	1.2730952	1.062417	1.525551	0.0088982
MCM7	1.3003088	1.0803385	1.5650678	0.005483
SLC2A2	0.8952337	0.8067341	0.9934418	0.037173
APOC1	0.8154871	0.7078436	0.9395002	0.0047428
HIF1A	1.2692792	1.0289702	1.5657106	0.0259711
GLS	1.3315622	1.0565427	1.6781695	0.0152692
TFR2	0.8716248	0.7734674	0.982239	0.0241995
SORD	0.7950769	0.6646398	0.9511126	0.0121351
DSG2	1.3323723	1.12945	1.5717525	0.0006642
AGXT	0.8819124	0.8030537	0.9685149	0.0085549
ANXA5	1.3584106	1.1000949	1.677382	0.0044212
PFKP	1.2746883	1.0728315	1.5145251	0.0057943
MMP14	1.1757901	1.0072703	1.3725039	0.0401946
LRRC1	1.2719373	1.005861	1.6083978	0.0445619
ITGA5	1.3926694	1.0940755	1.772755	0.0071405
BAAT	0.8698249	0.7705453	0.9818961	0.0241065
F13B	0.8493502	0.7445365	0.9689192	0.0151065
RASSF3	1.2802845	1.025099	1.5989951	0.0293665
OLFML2B	1.284346	1.0555845	1.5626837	0.0124028
CYP2C9	0.8814531	0.8068864	0.9629107	0.0051415
CTSC	1.3550202	1.0918824	1.6815727	0.0058172
GPT	0.8462881	0.733248	0.9767548	0.0225201
RAP1GAP	1.2117564	1.0241499	1.4337292	0.0252201
STK39	1.234072	1.0179384	1.4960962	0.0322781
HMGB2	1.3995133	1.1374701	1.7219243	0.0014845
AFM	0.8638749	0.7829708	0.9531388	0.0035387
CDC25B	1.4182897	1.1528667	1.7448206	0.0009479
ALDH5A1	0.7689653	0.6242682	0.9472014	0.0135131
ADH1A	0.8940065	0.8172984	0.9779141	0.0143689
SELENBP1	0.8138777	0.6865689	0.9647931	0.0176473
DAB2	1.3849927	1.148908	1.6695894	0.000636
HSD17B8	0.7752382	0.6097501	0.9856403	0.0377072
FOXJ1	1.2165261	1.0041195	1.4738642	0.0452897
FBLN1	1.1798123	1.0272203	1.3550717	0.0192827
LAPTM4B	1.3038893	1.1176255	1.5211959	0.000741
C6	0.8596159	0.7653518	0.96549	0.0106927
GNMT	0.8906126	0.8090269	0.9804256	0.018116
ITGAV	1.2919072	1.0618986	1.5717359	0.0104567

SCRN1	1.2478939	1.0345904	1.5051748	0.0205845
CA5A	0.8125634	0.6900032	0.9568931	0.0128396
C4BPB	0.8584149	0.7575848	0.972665	0.0166338
C4BPA	0.9076659	0.832549	0.9895602	0.0279436
TMPRSS6	0.8712982	0.7756565	0.9787329	0.0202159
RECQL4	1.3053292	1.0908665	1.5619551	0.0036177
LECT2	0.8783865	0.7890178	0.9778777	0.0178556
IER3	1.2010072	1.0258069	1.4061306	0.0228058
CSF1	1.3638848	1.1146553	1.6688404	0.0025762
F11	0.837172	0.7126788	0.9834122	0.0304955
CPS1	0.9144623	0.8459263	0.9885509	0.0244699
PTK7	1.3279212	1.0857522	1.6241041	0.0057651
FSCN1	1.2412639	1.0230287	1.5060535	0.0284706
IGFBP3	1.1651286	1.0095742	1.3446507	0.0365919
ALDOB	0.9129147	0.8413911	0.9905183	0.028609
MMP9	1.1625411	1.0177556	1.3279238	0.0264658
PFN2	1.3396454	1.1588865	1.5485985	7.69E-05
GPLD1	0.8507862	0.7353292	0.9843715	0.0298819
CAPG	1.1888169	1.0309731	1.3708268	0.0173296
SERPINE2	1.2448637	1.0410551	1.488572	0.016349
RORC	0.8200223	0.7048626	0.9539967	0.0101721
HMGCS2	0.8742249	0.7919445	0.965054	0.0076923
ATP1A1	1.1873423	1.0108158	1.3946969	0.0365318
FTCD	0.8647201	0.7780602	0.9610321	0.0069826
ALDH8A1	0.8495542	0.7430885	0.9712736	0.0170042
G6PC	0.8789384	0.7985325	0.9674406	0.0083841
CYP7A1	0.8950934	0.8129917	0.9854863	0.0239576
F7	0.8276571	0.7009826	0.9772229	0.0256272
PRAME	1.1925823	1.0478138	1.3573523	0.007646
CHAD	0.7913995	0.6502145	0.9632408	0.0196208
SLC25A15	0.8414981	0.7120438	0.9944881	0.0428864
TREM2	1.1936051	1.020912	1.3955102	0.0264513
BHMT2	0.8545296	0.7353425	0.9930348	0.0402512
VEGFB	1.1660418	1.0087526	1.3478561	0.0377241
EPS8L3	1.1592215	1.0067829	1.334741	0.0399816
C1S	0.7719034	0.6522066	0.9135675	0.0025999
PHLDA2	1.2272795	1.0712431	1.406044	0.0031582
FCER1G	1.2647005	1.0687489	1.4965791	0.006256
PROZ	0.8290149	0.7170967	0.9584003	0.0112708
UCHL1	1.1220123	1.0152785	1.2399668	0.0239913
RAMP1	0.87564	0.7807493	0.9820636	0.023254
GAMT	0.8190717	0.6824297	0.9830735	0.0320898
IGF2BP2	1.2058134	1.029024	1.4129757	0.0206865
FGA	0.8957273	0.8159159	0.9833458	0.0207406
BAIAP2L2	1.1923344	1.0323634	1.3770939	0.016698
OGDHL	0.844247	0.7309083	0.9751605	0.0213381
GADD45G	0.8419357	0.7242522	0.9787416	0.0251118
BAMBI	1.2015082	1.0335145	1.3968087	0.0168966
UGT2B15	0.8794629	0.8096429	0.9553039	0.0023391
SPP1	1.1423757	1.0691049	1.2206681	8.30E-05
SCPEP1	1.1815444	1.0002231	1.3957357	0.049694

RARRES2	0.7725724	0.6666139	0.895373	0.0006073
CFHR5	0.8972646	0.8218514	0.9795978	0.0155137
CPVL	1.2837009	1.0702065	1.5397851	0.0071221
SLC17A2	0.8351713	0.7316706	0.9533129	0.0076249
USH1C	1.1975056	1.0318023	1.3898202	0.0176938
LCAT	0.7895701	0.680416	0.9162349	0.0018558
S100A9	1.2187254	1.0937004	1.3580425	0.0003412
AKR1D1	0.8914929	0.7991539	0.9945013	0.0395135
FGG	0.9091311	0.8267375	0.9997361	0.0493669
FGB	0.9089001	0.8310152	0.9940847	0.0366391
LGALS3	1.2341871	1.0741736	1.418037	0.0029791
SLC22A7	0.904491	0.8259359	0.9905175	0.0303495
MSC	1.1702119	1.0498717	1.304346	0.0045259
VNN2	1.2694348	1.112395	1.4486444	0.0003988
SERPINE1	1.1669581	1.042463	1.3063209	0.0073084
HGFAC	0.9111568	0.8370097	0.9918722	0.0316811
SFN	1.136193	1.0358495	1.2462569	0.0067979
NQO1	1.0893663	1.0100284	1.1749363	0.0265141
