# Supplementary figures and images for: Integrating bulk RNA-seq and ScRNA-seq to identify manganese metabolism-related subtypes and immunoregulatory mechanisms in liver hepatocellular carcinoma
Source: Open Life Sci. 2026 Apr 29;21(1):20251298. doi: 10.1515/biol-2025-1298 (PMC13127686; doi:10.1515/biol-2025-1298)

A

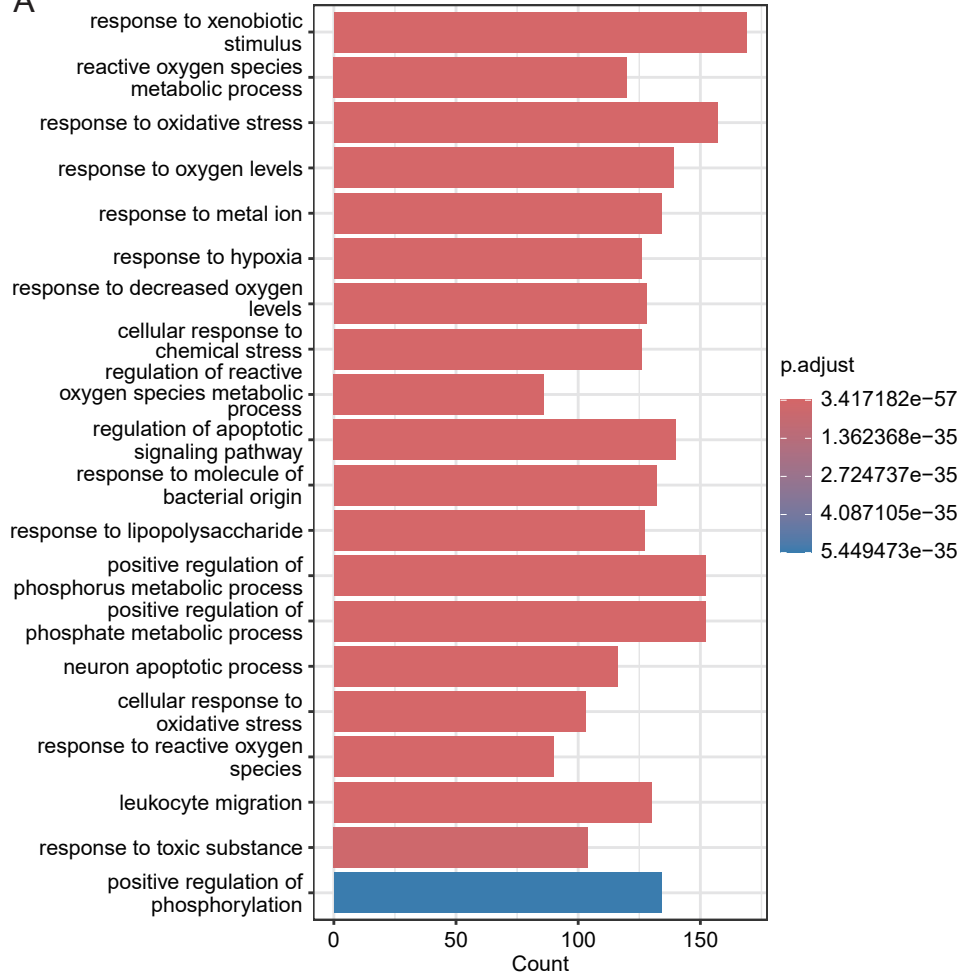

B

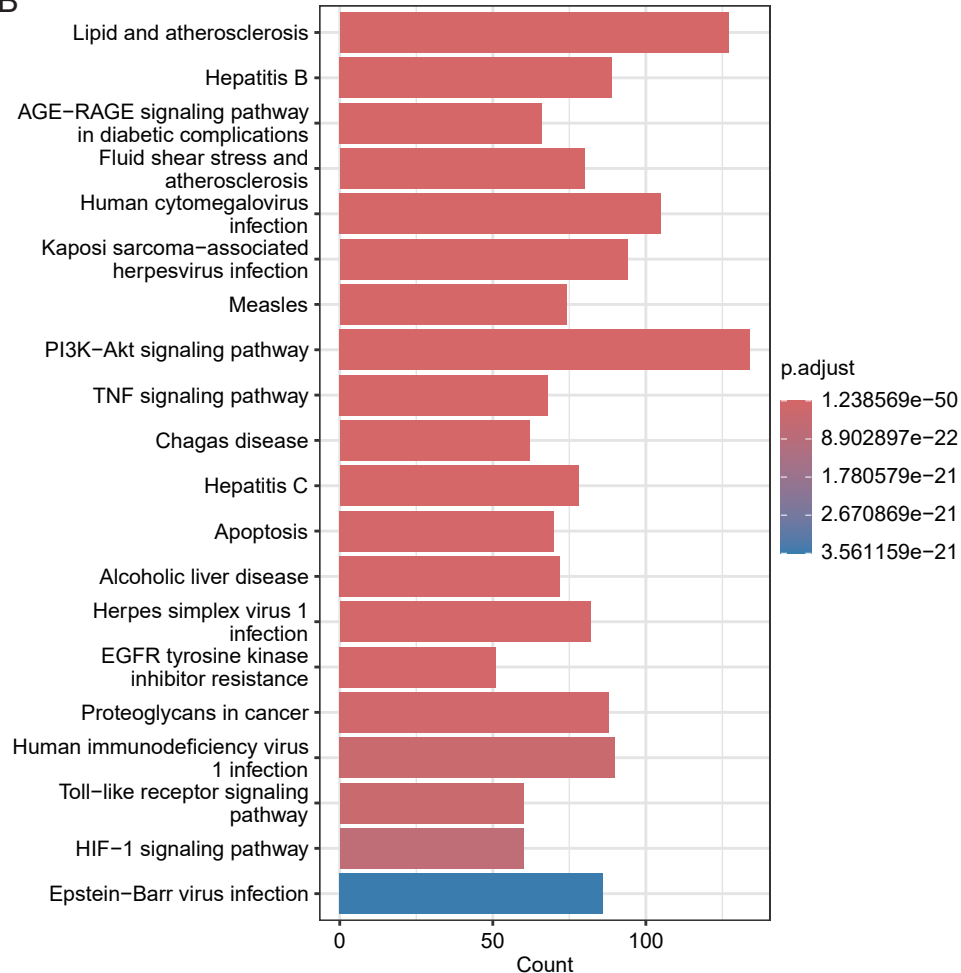

Supplement: Supplementary file 2 — Supplementary Material [file j_biol-2025-1298_suppl_002.pdf]
