# Supplementary material for: Integrating bulk RNA-seq and ScRNA-seq to identify manganese metabolism-related subtypes and immunoregulatory mechanisms in liver hepatocellular carcinoma
Source: Open Life Sci. 2026 Apr 29;21(1):20251298. doi: 10.1515/biol-2025-1298 (PMC13127686; doi:10.1515/biol-2025-1298)
Supplement: Supplementary file 3 — Supplementary Material [file j_biol-2025-1298_suppl_003.pdf]

Supplementary Figure 1: GO and KEGG enrichment analysis of manganese metabolism-related genes (MMRGs).

Supplementary Table 1: Univariate regression analysis results（$p<0.05$）
